# Regulators of plant biomass degradation in ascomycetous fungi

**DOI:** 10.1186/s13068-017-0841-x

**Published:** 2017-06-12

**Authors:** Tiziano Benocci, Maria Victoria Aguilar-Pontes, Miaomiao Zhou, Bernhard Seiboth, Ronald P. de Vries

**Affiliations:** 1 Fungal Physiology, Westerdijk Fungal Biodiversity Institute & Fungal Molecular Physiology, Utrecht University, Uppsalalaan 8, 3584 CT Utrecht, The Netherlands; 2 Research Area Biochemical Technology, Institute of Chemical and Biological Engineering, TU Wien, 1060 Vienna, Austria

**Keywords:** Plant biomass degradation, Transcription regulation, CAZy genes, Fungi, Bioeconomy

## Abstract

Fungi play a major role in the global carbon cycle because of their ability to utilize plant biomass (polysaccharides, proteins, and lignin) as carbon source. Due to the complexity and heterogenic composition of plant biomass, fungi need to produce a broad range of degrading enzymes, matching the composition of (part of) the prevalent substrate. This process is dependent on a network of regulators that not only control the extracellular enzymes that degrade the biomass, but also the metabolic pathways needed to metabolize the resulting monomers. This review will summarize the current knowledge on regulation of plant biomass utilization in fungi and compare the differences between fungal species, focusing in particular on the presence or absence of the regulators involved in this process.

**Electronic supplementary material:**

The online version of this article (doi:10.1186/s13068-017-0841-x) contains supplementary material, which is available to authorized users.

## Background

Plant biomass is the most abundant carbon source on Earth and therefore is of major importance for ecology and the global carbon cycle. Fungi are highly efficient degraders of plant biomass. Due to the complexity and heterogeneity of plant biomass (see “Plant biomass composition and its degradation”) (Table 1), fungi have developed a complex and efficient degrading machinery, consisting of a large set of hydrolytic and oxidative enzymes. These enzymes are not only involved in saprobic degradation of plant biomass, but also in other types of plant–fungal interaction, such as pathogenicity and symbiosis (mycorrhizae) or parasitism.

**Table 1:** Composition of plant biomass polymers. Based on Kowalczyk et al. [37]

Polymer type	Polymer	Monomers
Cellulose		d-Glucose
Hemicellulose	Xylan	d-Xylose
	Glucuronoxylan	d-Glucuronic acid, d-xylose
	Arabinoglucuronoxylan	d-Xylose, l-arabinose
	Arabinoxylan	d-Xylose, l-arabinose
	Galacto(gluco)mannan	d-Glucose, d-mannose, d-galactose
	Mannan/galactomannan	d-Mannose, d-galactose
	Xyloglucan	d-Glucose, d-xylose, d-fructose, d-galactose
	β(1,3)/(1,4)-glucan	d-Glucose
Pectin	Homogalacturonan	d-Galacturonic acid
	Xylogalacturonan	d-Galacturonic acid, d-xylose
	Rhamnogalacturonan I	d-Galacturonic acid, l-rhamnose, d-galactose, l-arabinose, ferulic acid, d-glucuronic acid
	Rhamnogalacturonan II	d-Galacturonic acid, l-rhamnose, d-galactose, l-arabinose, l-fucose, d-glucose, d-manno-octulosonic acid (KDO), d-lyxo-heptulosaric acid (DhA), d-xylose, d-apiose, l-acetic acid
Inulin		d-Fructose, d-glucose
Starch	Amylose	d-Glucose
	Amylopectin	d-Glucose
Various gums		d-Galacturonic acid, l-rhamnose, d-galactose, l-arabinose, d-xylose, l-fucose (depending on the specific gum type)
Lignin		Monolignols: ρ-coumaryl alcohol, coniferyl alcohol, sinapyl alcohol

However, plant biomass is also the source of many food and industrial products that are used in our society. This has resulted in a broad interest from researchers and industry in the enzymatic degradation of plant biomass, starting with the first application of these degrading enzymes in the beginning of the last century (e.g., hydrolysis of starch and maltose [1]). Since then many applications have been developed in several industrial fields, such as food and feed, pulp and paper, detergents, textile, crop protection, and more recently biofuels and biochemicals [2, 3].

Currently, only a small number of Ascomycetes (including *Aspergillus* spp. and *Trichoderma reesei*) have been developed for industrial applications [4], although a larger group is being used as a source of novel enzymes. The major companies producing these fungal enzymes cocktails, such as Novozymes (http://www.novozymes.com), DSM (http://www.dsm.com), Roal Oy (http://www.roal.fi), AB Enzymes (http://www.abenzymes.com), and DuPont (http://www.dupont.com), have selected these fungi, due to their good fermentation properties and high level of protein production. However, enzymatic hydrolysis (i.e., saccharification) is still one of the major bottlenecks in the biorefinery process, due to the recalcitrance of plant cell wall and the insufficient efficiency of current enzymatic cocktails to convert all the biomass into the desired products. Strategic improvements are required, and recent developments in fungal research (genomics, transcriptomics and proteomics) have provided a much deeper insight into the mechanisms/strategies of fungi related to plant biomass utilization.

In general, regulation of fungal gene expression related to plant biomass utilization occurs by activation of gene expression via specific inducers (see “Induction of plant biomass utilization”) in balance with repression of gene expression via carbon catabolite repression (CCR) (see “Carbon catabolite repression: CreA/1”). The sensing of inducers starts a signaling pathway resulting in the activation of transcriptional regulators, which is followed by the production of plant biomass-degrading enzymes as well as the metabolic pathways to use the accumulating sugar monomers [5].

Several transcription factors (TFs) involved in plant biomass have been characterized from the fungal kingdom, mainly belonging to zinc binuclear cluster family [6] (see “Transcription factors directly involved in plant biomass degradation”) (Table 2). However, only a few regulators are conserved across the fungal kingdom, such as the carbon catabolite repressor CreA, while the majority of them are restricted to subgroups of fungal species [6] (Additional file 1). The presence of a regulator in a larger group of fungi also does not guarantee that its function is fully conserved: a phenomenon known as “transcriptional rewiring” [7]. Differences in target genes and induction have been observed, such as for the well-studied case of XlnR (see “The (hemi-)cellulolytic regulator XlnR/XYR1/XLR1”) [8] or Gal4 in yeasts [9]. Evolution of fungal TFs appears to be ruled mainly by transcriptional rewiring and divergence as consequence of duplication or horizontal gene transfer (also from bacteria and viruses), while parallel or convergent evolution appears to be relatively rare [10–12]. In addition, TFs need also to have access to chromatin (which is controlled by other factors, such as methylation, acetylation, and histone modification) [13–15]. This adds a second level of complexity in gene regulation ruled by chromatin access and epigenetics.

**Table 2:** Comparison of transcription factors involved in plant biomass utilization

TF	Class	Binding site	Function		Sections	Fungi	References
			Main	Secondary			
XlnR/XLR1/XYR1	Zn_2_Cys_6_	GGCTRRR or GGC(A/T)_3_	(Hemi)-cellulose utilization	Unknown	“The (hemi-)cellulolytic regulator XlnR/XYR1/XLR1”	*Aspergillus* spp., *Trichoderma* spp., *Fusarium* spp., *N. crassa*, *M. oryzae*, *P. canescens*, *T. cellulolyticus*	[8, 84*]
AraR	Zn_2_Cys_6_	Unknown	l-Arabinose utilization	Unknown	“l-Arabinose-responsive regulators (AraR and ARA1)”	*Aspergillus* spp.	[55, 112*]
ARA1	Zn_2_Cys_6_	Unknown	l-Arabinose utilization	Unknown	“l-Arabinose-responsive regulators (AraR and ARA1)”	*Magnaporthe oryzae*	[102*]
CLR-1/ClrA	Zn_2_Cys_6_	CGGN_5_CGGNCCG	Cellulose utilization	Unknown	“*Neurospora crassa* cellulose regulators CLR-1 and CLR-2 and their homologs”	*N. crassa, Aspergillus* spp.	[98*, 114, 119]
CLR-2/ClrB/ManR	Zn_2_Cys_6_	CGGN_11_CGG or YAGAAT	Cellulose utilization	Unknown	“*Neurospora crassa* cellulose regulators CLR-1 and CLR-2 and their homologs”	*N. crassa*, *Aspergillus* spp., *T. cellulolyticus*, *P. oxalicum*	[98*, 114, 119]
ACE2	Zn_2_Cys_6_	GGCTAATAA or GGC(T/A)_4_ or XAE	Cellulose utilization	Unknown	“Specific activators of cellulase gene expression in *T. reesei*: ACE2 and ACE3”	*Trichoderma reesei*	[126]*
ACE3	Zn_2_Cys_6_	Unknown	Cellulose utilization	Unknown	“Specific activators of cellulase gene expression in *T. reesei*: ACE2 and ACE3”	*Trichoderma reesei*	[121]*
AmyR	Zn_2_Cys_6_	CGGN_8_(C/A)GG or CGGAAATTTAA	Starch utilization	Unknown	“Amylolytic regulation: AmyR and MalR”	*Aspergillus* spp.	[129–131]
MalR	Zn_2_Cys_6_	Unknown	Maltose utilization	Unknown	“Amylolytic regulation: AmyR and MalR”	*Aspergillus* spp.	[128, 137*]
BglR/COL-26	Zn_2_Cys_6_	Unknown	Sugar sensing	BGL repressor	“Glucose-sensing regulator: BglR and COL-26”	*Trichoderma reesei, Neurospora crassa*	[99, 118, 139*]
ClbR	Zn_2_Cys_6_	CGG OR CCG	Cellobiose utilization	Unknown	“ClbR: cellobiose response regulator”	*Aspergillius aculeatus*	[17, 140*]
RhaR	Zn_2_Cys_6_	Unknown	l-Rhamnose utilization	Unknown	“Pectinolytic regulation: RhaR, GaaR, AraR and GaaX”	*Aspergillus* spp.	[42, 144*]
GaaR	Zn_2_Cys_6_	TCCNCCAAT	Galacturonic acid utilization	Unknown	“Pectinolytic regulation: RhaR, GaaR, AraR and GaaX”	*Botrytis cinerea*, *Aspergillus niger*	[146, 147*]
InuR	Zn_2_Cys_6_	CGGN_8_CGG	Inulin utilization	Unknown	“Inulinolytic regulation: InuR”	*Aspergillus niger*	[37, 148*]
GalX	Zn_2_Cys_6_	Unknown	d-Galactose utilization	Unknown	“d-Galactose-responsive regulators: GalR and GalX”	*Aspergillus* spp.	[152*]
GalR	Zn_2_Cys_6_	Unknown	d-Galactose utilization	Unknown	“d-Galactose-responsive regulators: GalR and GalX”	*Aspergillus nidulans*	[113, 149*]
GaaX	Unknown	Unknown	Galacturonic acid repressor	Unknown	“Pectinolytic regulation: RhaR, GaaR, AraR and GaaX”	*Aspergillus niger*	[146]
CreA/CRE1	Cys_2_His_2_	SYGGRG	Carbon catabolite repression	Unknown	“Carbon catabolite repression: CreA/1”	*Aspergillus* spp., *Trichoderma* spp., *N. crassa*, etc.	[156, 157*]
ACE1	Cys_2_His_2_	Unclear	Cellulase repression	Unknown	“Activator of cellulase expression 1 in *T. reesei* and hemicellulase regulator 1 in *N. crassa*”	*Trichoderma reesei*	[125, 198]
HCR-1	Cys_2_His_2_	Unknown	Hemicellulase repressor	Unknown	“Activator of cellulase expression 1 in *T. reesei* and hemicellulase regulator 1 in *N. crassa*”	*Neurospora crassa*	[199]
McmA	MADS-box	CC(A/T)_6_GG	Cellulase regulation	Unknown	“McmA”	*Aspergillus nidulans*	[124*]
XPP1	E-box–HLH	WCTAGW + AGAA	1°–2° metabolism switch	Xylanases repressor	“Xylanase promoter-binding protein (XPP1)”	*Trichoderma reesei*	[206, 207]
VIB1	p53-like	Unknown	C-derepression	CLR-2 induction, cellulases induction	“The (hemi-)cellulolytic regulator XlnR/XYR1/XLR1”	*Neurospora crassa*	[99]
HAP complex	CBF	CCAAT	Chromatin remodeling, respiratory metabolism	CAZy regulation	“Chromatin access”	*Aspergillus* spp., *N. crassa*, *Trichoderma* spp., etc.	[213–216]
WC-1/BLR1	GATA	WGATAR	Blue light/UV-A response, circadian rhythms	1° metabolism response, (hemi)-cellulose utilization	“Light effect”	*Neurospora crassa, Trichoderma* spp.	[221, 235, 241]
WC-2/BLR2	GATA	WGATAR	Blue light/UV-A response, circadian rhythms	1° metabolism response, (hemi)-cellulose utilization	“Light effect”	*Neurospora crassa*, *Trichoderma* spp.	[221, 234, 235]
VeA/VEL1	Velvet	Unknown	Light response	2° metabolism response, (a)sexual development	“Light effect”	*Aspergillus* spp., *Trichoderma* spp., *N. crassa, Fusarium* spp., etc.	[204, 205, 224]
VelB	Velvet	Unknown	Light response	2° metabolism	“Light effect”	*Aspergillus* spp.	[212, 224]
AreA/NIR2	Cys_2_Cys_2_	HGATAR	*N*-assimilation/sensing	Chromatin remodeling	“TFs involved in nitrogen and pH regulation”	*Neurospora crassa, Aspergillus* spp.	[261, 262, 267]
AreB	GATA	Unknown	Nitrogen metabolite repression	Morphology and asexual development	“TFs involved in nitrogen and pH regulation”	*Colletotrichum gloeosporioides*, *Aspergillus* spp.	[258, 267, 268]
NmrA/1	Rossmann fold	Unknown	Nitrogen metabolite repression	Unknown	“TFs involved in nitrogen and pH regulation”	*Aspergillus* spp., *Neurospora crassa*	[260, 266, 267]
NirA/NIT-4	Zn_2_Cys_6_	Unknown	Nitrate pathway	Unknown	“TFs involved in nitrogen and pH regulation”	*Aspergillus* spp., *Neurospora crassa*	[263, 264]
PacC/1	Cys_2_His_2_	GCCARG	Alkaline pH response	Unknown	“TFs involved in nitrogen and pH regulation”	*Aspergillus* spp., *T. reesei*, *N. crassa*	[271, 276, 283]
PacX	Zn_2_Cys_6_	Unknown	PacC repressor	Unknown	“TFs involved in nitrogen and pH regulation”	*Aspergillus nidulans*	[282]

References reported here are the most relevant (for more see, specific sections). References with* are the first characterization in fungi and selected as protein reference for orthologous clustering searching

Regulation of gene expression directly affects the composition of the resulting enzyme mixtures and is therefore highly relevant for applications [16], resulting in detailed studies in several species, such as *Aspergillus* spp. [17–19], *T. reesei* [20–23], *Neurospora crassa* [24], and *Penicillium oxalicum* [25, 26]. This review will discuss the knowledge of the main ascomycetous transcriptional regulators directly involved in plant biomass utilization, focusing in particular on the regulatory differences among Ascomycetes. In addition, we will summarize the knowledge about regulatory factors indirectly involved in plant biomass utilization.

## Plant biomass composition and its degradation

Plant biomass consists mainly of polysaccharides, but also contains proteins and the aromatic polymer lignin. Its precise composition is highly complex and varies depending on plant species and tissue, season, and geographic location. Plant polysaccharides (Table 1) can be divided into plant cell wall polysaccharides (e.g., cellulose, hemicelluloses, pectins) and storage polysaccharides (e.g., starch, inulin, gums) [5]. They consist of many monomeric components that are attached to each other through a variety of linkages. Plant cell wall polysaccharides are not only linked to each other, but also to the aromatic polymer lignin, providing the main strength and structure for the plant cell wall, and serving as a defense against microbial attack.

Cellulose is the most abundant plant cell wall polysaccharide, consisting of a linear chain of β-1,4-linked d-glucose residues, organized in bundles called microfibrils [27]. Hemicelluloses are more diverse in nature, and they are classified in three main types, depending on the backbone: xylan (β-1,4-linked d-xylose), xyloglucan (β-1,4-linked d-glucose), and mannan (β-1,4-linked d-mannose). These backbones are interrupted, branched, or decorated with several different monomers or chains, resulting in different variants of these polymers [28, 29]. Pectin is the third polysaccharide in plant cell wall, but its amount depends on the plant species and tissue. This polysaccharide consists of four substructures with galacturonic acid as the main monomeric component: homogalacturonan (HGA), xylogalacturonan (XGA), and the more complex rhamnogalacturonan I (RG-I) and II (RG-II) [2].

Starch is one of the main storage polysaccharides, consisting of an α-1,4-linked polymer (amylose) of d-glucose residues which can be branched at α-1,6-linked points (amylopectin) [30, 31]. Another major storage polysaccharide is inulin that consists of a branched β-2,1-linked chain of d-fructose with a terminal d-glucose residue [32, 33]. Gums are another varied group of storage polysaccharides, containing many structures. Some of them (e.g., locust bean gum, guar gum) are similar to cell wall galactomannan [2, 34].

Lignin is the most complex and recalcitrant heteropolymer, consisting of aromatic alcohols known as monolignols, built from three phenylpropanoid precursors: ρ-coumaryl alcohol, coniferyl alcohol, and sinapyl alcohol. The exact structure of lignin varies depending on plant species or tissue [2, 35].

In order to release the monomers present in those complex structures, the simultaneous action of several plant biomass-degrading enzymes are required.

Those enzymes have been classified into families in the Carbohydrate-Active enZymes database (CAZy) [36] (http://www.cazy.org), based on their amino acid sequences. CAZy contains six main groups: glycoside hydrolases (GH), glycosyltransferases (GT), polysaccharide lyases (PL), carbohydrate esterases (CE), auxiliary activities (AA). and carbohydrate-binding modules (CBMs). In each group, there are several families, some containing only a single known enzyme activity (e.g., GH67), while others contain several activities (e.g., GH43) and are therefore not an immediate indication of enzyme function [2].

## Induction of plant biomass utilization

In order to utilize plant biomass, fungi need to recognize its components and subsequently induce the production of plant biomass-degrading enzymes, as well as the metabolic pathways needed to convert these sugars into energy and/or bioproducts. These signal compounds are commonly defined as inducer, but polysaccharides are too large to enter fungal cells and therefore probably cannot act directly as inducers. Fungi likely recognize the presence of complex polymers through low molecular weight compounds derived from them, such as monosaccharides or disaccharides. As these simple compounds are rare in the environment, their presence is an indication for the fungus that specific polysaccharides might be present. When one of these simple compounds is sensed by the fungus, a signaling pathway results in the activation of one or more transcriptional activators (see “Transcription factors directly involved in plant biomass degradation”, “Other factors affecting plant biomass utilization”), which (in most of the cases) enters the nucleus and triggers the expression of its target genes, encoding hydrolytic/oxidative enzymes (CAZymes), as well as the metabolic pathways needed to utilize the available C-source [37].

The main theory is that inducers are produced by hydrolysis of polysaccharides by small amounts of constitutively produced enzymes. These degradation products might then be further modified by transglycosylation [38–40]. According to an integrating hypothesis, proposed more than one decade ago, some genes encoding plant biomass-degrading enzymes are induced by carbon starvation, acting as scouting enzymes playing a foraging role under these conditions. They release the inducing molecules that trigger the main hydrolytic response [41]. These phenomena have been observed in *Aspergillus niger* in response to pectin [42], starch [43], and wheat straw [44].

Different inducers for (hemi-)cellulases have been described in fungi: d-xylose in *A. niger* [45], cellobiose in *Aspergillus oryzae* [46], gentiobiose in *Penicillium purpurogenum* [47], and sophorose in *Aspergillus terreus* and *T. reesei* [48, 49]. Amore et al. [50] have summarized all known inducers and related induced genes in the three model fungi *A. niger*, *T. reesei*, and *N. crassa*. Some of the more interesting cases are discussed below. However, in the majority of the cases, the exact structure of the inducer is unknown, and “inducer” is often used to refer to the mono- or disaccharide which triggers the initial transcription of the target genes.

Commonly, it is believed that in *Aspergillus* spp., the (hemi)-cellulolytic systems are strictly coregulated via the monomeric inducer d-xylose [51–53] and/or cellulose-derived disaccharides [46, 53], while the respective systems in *T. reesei* and *N. crassa* are more distinctively regulated. It has been shown that l-arabitol is the inducer of the arabinanolytic and xylanolytic system in Aspergilli [54–56], while in *T. reesei*, only l-arabinose and not l-arabitol can trigger these effects [57]. It has been demonstrated in *A. niger* [58, 59] and *T. reesei* [57, 60] that d-xylose induction is concentration-dependent: at very low concentrations, it acts as an inducer for xylanases, while at higher concentrations as a repressor through CreA/Cre1.

In *T. reesei*, pure saccharides such as d-xylose, xylobiose, sophorose, lactose, and galactose can act as inducers for (hemi)-cellulase production, but each one can activate only part of these systems [50, 61, 62]. Therefore, induction of (hemi-)cellulases in *T. reesei* appears different and more diverse than in other fungi [57], such as *N. crassa* or *A. niger,* and a general model for the substrates recognition and the induction of (hemi)-cellulolytic systems in *T. reesei* has been suggested [63]. While every fungus appears to have a best (natural) inducer for (hemi-)cellulase production, such as d-xylose in *Aspergillus,* sophorose in *T. reesei* [64] or cellobiose in *N. crassa* [65], other compounds can also have an inducing effect. Some examples of this are d-galactose in *N. crassa* and cellobiose and lactose in *T. reesei* [66, 67].

At the industrial level, lactose is a promising inducer for (hemi)-cellulase production in *T. reesei* [67], because it is the only soluble and also cheap inducer of carbon source ever shown, at least in this fungus.

Although the exact mechanism of this induction is not known yet, *xyr1* is involved in this process [68, 69]. In contrast, the induction of pectinolytic genes is more complex: some of them are constitutively expressed, while others are specifically induced, as studied in Aspergilli [42, 70, 71]. These constitutively expressed genes probably encode the scouting enzymes that are needed to liberate some monomeric sugars from pectin, such as galacturonic acid, rhamnose, arabinose, galactose, or xylose, which induce the other pectinolytic genes through specific regulatory systems [70, 71].

The degradation of crude plant biomass is more complex than that of simple polysaccharides, requiring a network of TFs which respond to different inducers. These regulators need to act together in a coordinated manner to express the right enzymes over time, depending on the substrate and the fungal species. In the literature, only few studies have covered these aspects, but recently, expression profiles of a “large” time course of CAZy and pathway genes have been reported in Aspergilli [72–74], *Trichoderma reesei* [75], *Neurospora crassa* [39], and *Myceliophthora thermophila* [76] or mixed cultures [77].

As de Souza et al. [78] have shown, *A. niger* during growth on steam-exploded sugarcane bagasse (SEB) prefers to use first d-glucose, followed by d-xylose, and finally l-arabinose, affecting enzyme production over time. In this system, the transcription factor XlnR (xylanolytic regulator, see “The (hemi-)cellulolytic regulator XlnR/XYR1/XLR1”) has been confirmed as the main regulator for the expression of genes encoding complex substrates-degrading enzymes, overruling effects of AraR (arabinanolytic regulator; see “l-Arabinose-responsive regulators (AraR and ARA1)”). Surprisingly and in contrast to previous reports, XlnR and AraR have an overlapping role in promoting the expression of some degrading enzyme-encoding genes depending on the substrate (SEB or monosaccharides mix), such as *bglA/4* (β-glucosidases), *agdA/B* (α-glucosidases)*, eglA* (endoglucanase), *lacA* (α-galactosidase), *aglB* (β-galactosidase), *axhA* (arabinoxylan arabinofuranohydrolase). and *xlnD* (β-endoxylanase) [78]. Interestingly, Alazi et al. [79] showed that some pectinolytic genes are more expressed at late time points, suggesting that they are induced by starvation or derepressed conditions. In addition, Daly et al. [74] showed that the type of pretreatment of the plant biomass (wheat straw in this case) will also affect the expression of CAZy genes over time, reflecting the accessibility/availability of saccharides/polymers due to the different pretreatments.

All these data suggest that the presence of a fine-tuned and complex crosstalk between inducers and regulatory systems involved in plant biomass utilization in filamentous fungi still remains to be fully understood.

## Transcription factors directly involved in plant biomass degradation

Many fungal transcription factors have been described to be directly involved in the regulation of plant biomass utilization (Additional file 1). Their number has increased rapidly in the last years, due to deeper studies and novel tools (“Omics” Era) (i.e., transcriptomics and bioinformatics), providing new insights in this field.

So far, the majority of these TFs belong to the zinc cluster family. This TF family is characterized by the presence of zinc finger(s) in their binding domains, and it is classified in “fold groups” based on the overall shape of the protein backbone in the folded domain, which depends and on the amounts of cysteine and histidine residues. The majority of positive regulators appear to belong to the Zn_2_Cys_6_ class, while the repressors to the Cys_2_His_2_ class.

**Fig. 1:**
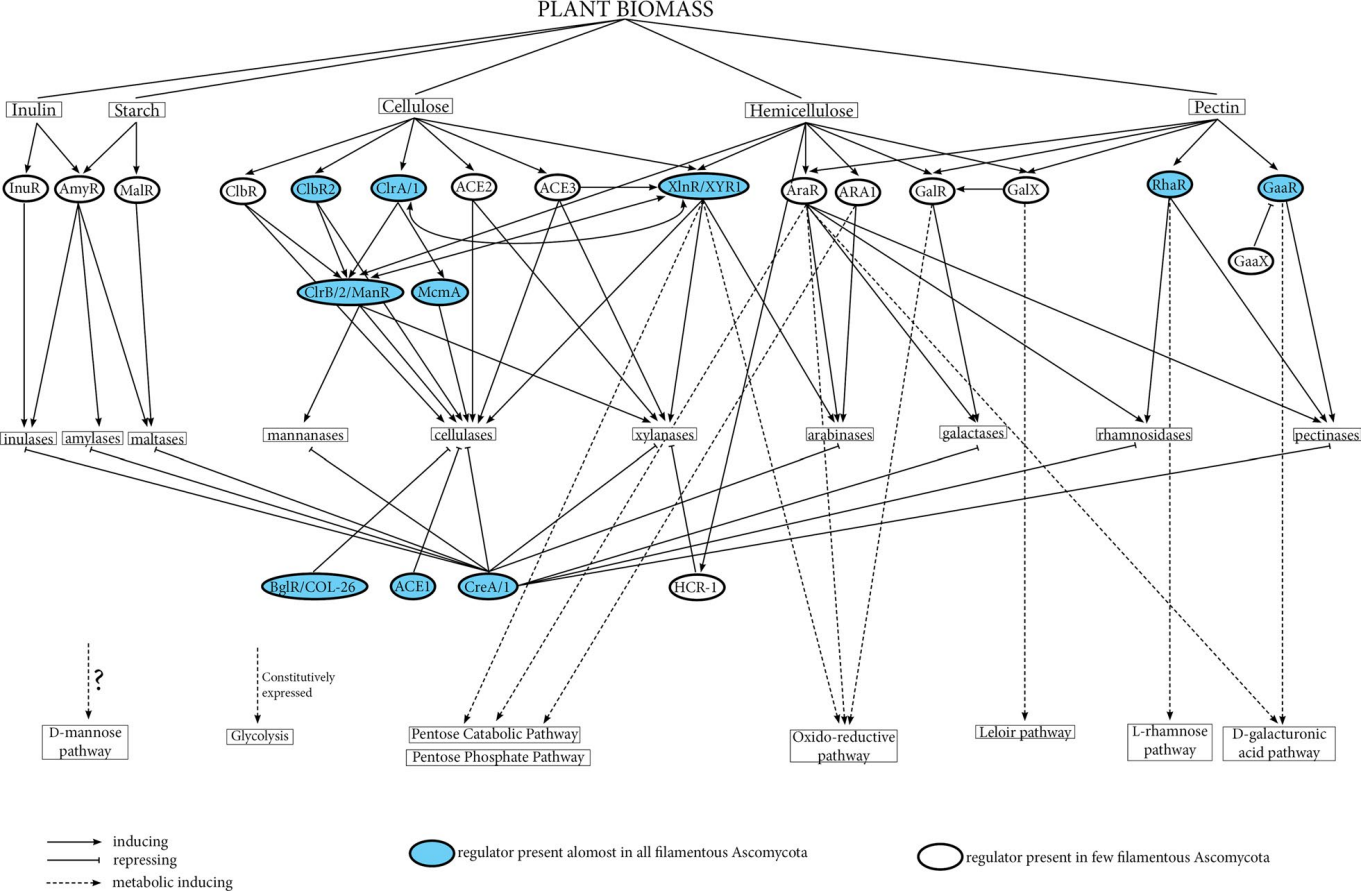
Overview of the regulators-network involved in plant biomass degradation. It is based on (and combining) the current knowledge of the model system *Aspergillus*, *Neurospora crassa* and *Trichoderma reesi* (This schema needs to be adapted to every fungal species, regarding the presence/absence, function(s). and interaction of/with each regulators)

Interestingly, several TFs are involved in cellulose degradation (CLR-1/2, XlnR, ACE2/3, ClbR, and McmA), suggesting overlapping function and/or fine tune regulation, depending on the species. Table 2 wants to give an overview to the known TFs and their role in carbon utilization although differences have been observed between fungal species. Figure 1 represents the network of these regulators, based on (and combining) the current knowledge of model systems, such as Aspergilli, *Neurospora crassa* and *Trichoderma reesei.* This schema is a general overview and needs to be adapted to every fungal species regarding the presence/absence, function(s). and possible interaction of each regulator.

Phylogenomic analysis was performed to study TF distribution along fungal kingdom following the pipeline of Todd et al. [6]. In summary, we used the same genome set to calculate genome scale protein ortholog clusters using OrthoMCL. The all-vs-all BlastP search required by OrthoMCL was carried out in a grid of 500 computers by parallel fashion. Clusters were detected according to [80], using inflction factor 1, E value cutoff 1 E^−3^, percentage match cutoff 60% as for identification of distant homologs [81]. Orthologs clusters based on already known TF (Table 2) were extracted and grouped according to their TF domains, such as Zn_2_Cys_6_, Cys_2_His_2_, MADS box, and basic helix-loop-helix (bHLH). Manual curation of the groups was performed by aligning the amino acid sequences with a suitable outgroup using MAFFT [82], and false positive were removed. Maximum likelihood trees (Additional files 2–5) were generated using MEGA5 [83] with 100 bootstraps and manually curated by refining the alignments (Additional file 1).

**Fig. 2:**
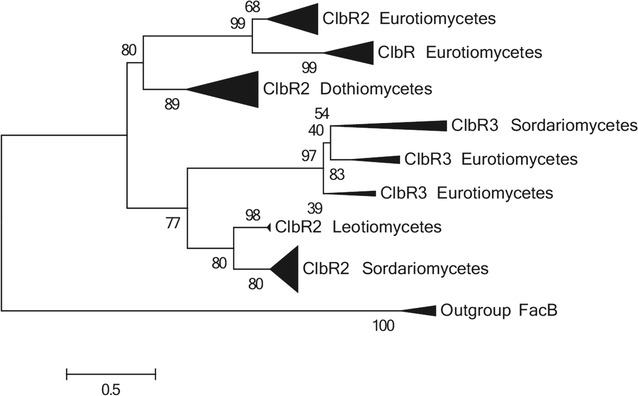
Maximum likelihood phylogenetic tree of ClbR paralogs

This approach allowed us to identify a new putative TF, named ClbR3 (paralog of ClbR/2) (see “ClbR: cellobiose response regulator”) (Fig. 2), which is present in just a few species (Additional file 2).

### Zn_2_Cys_6_ transcription factors

#### The (hemi-)cellulolytic regulator XlnR/XYR1/XLR1

*Aspergillus niger xlnR* was the first identified transcriptional factor [84] involved in the regulation of (hemi)-cellulose utilization and appears to be the main regulator for this process. Orthologs are present in nearly all filamentous Ascomycetes, confirming its key role in plant biomass utilization [6, 85]. This regulator has been well characterized in several fungi, such as *Aspergillus* spp. (*xlnR*) [46, 84, 86], *T. reesei* and *Trichoderma atroviride* (*xyr1*) [63, 87], *N. crassa* (*xlr*-*1*) [88], *F. graminearum* (*xyr1*) [89], *Fusarium oxysporum* (*xlnR*) [90], *Magnaporthe oryzae* (*xlr1*) [91], *Penicillium canescens* (*xlnR*) [92], and *Talaromyces cellulolyticus* (*xlnR*) [93], but its precise role differs depending on the species [8]. These differences include their set of target genes and binding efficiency, while the binding motif appears to be almost the same in all fungi, with the general consensus sequence GGCTRR (Table 2). In all fungi, this regulator controls d-xylose catabolism and the xylanolytic system [51, 63, 88–90], with the exception of *M. oryzae* [91] and *P. canescens* [92]. In *M. oryzae, xlr1* controls mainly d-xylose catabolism, while the xylanolytic system appears to be controlled not only by this regulator, but also by another unknown regulatory network [8]. The opposite appears to be the case in *P. canascens,* where XlnR is involved only in xylan degradation and not in d-xylose catabolism [92].

In Aspergilli [52, 86, 94, 95], except *Aspergillus aculeatus* [96], and *T. reesei* [63, 97] this regulator also controls the cellulolytic system, while this appears not to be the case in other species. So far, *T. reesei* is the only fungus with a clear XYR1 dependent regulation of the cellulolytic system [8]. In *N. crassa, xlr*-*1* can only modulate the expression levels of a few cellulases, suggesting that other TFs are more important [88] such as *clr*-*1* and *clr*-*2* [98] (see “*Neurospora crassa* cellulose regulators CLR-1 and CLR-2 and their homologs”). Interestingly in this fungus, XLR-1 induces also the TF VIB1, which represses glucose sensing and CCR (in a CRE-1-independent manner), under starvation inducing cellulase production by CLR-2 activation [99].

Moreover, *T. reesei* appears to be the only fungus that extended the role of *xyr1* also to regulating some genes of the arabinanolytic system. While *abf2* and *bxl1* are under its control, this is not the case for *abf1* and *abf3*. It is likely that another arabinanolytic transcription factor exists, that controls *abf1* and *abf3* [100, 101]. Recently, a new arabinose-responsive regulator was described in *M. oryzae*, that has orthologs only in Sordariomycetes and Leotiomycetes [102] (see “l-Arabinose-responsive regulators (AraR and ARA1)”).

Interestingly, regulation of the d-xylose reductase-encoding gene by XYR1 in *T. reesei* implicates control of XYR1 on d-galactose, lactose, l-arabinose, and d-xylose catabolism, due to the broad specificity and metabolic role of this enzyme [28, 63, 69, 100, 103].

Expression of most *xlnR/xyr1/xlr1* orthologs is not specifically induced [57, 58, 104, 105], but rather controlled by CCR (see “The (hemi-)cellulolytic regulator XlnR/XYR1/XLR1”). The only reported exception is *T. reesei*, where *xyr1* expression is clearly induced by lactose and cellulose [68, 69], but not during growth on xylan [106]. The mechanism by which *xyr1* responds to these inducers is still not fully understood, but appears to be linked to the nuclear import of XYR1 by the ß-importin KAP8 [107].

In *T. reesei*, the binding of XYR1 to his target genes appears to be more complex than in *Aspergillus* spp. [94]. XYR1 binds not only to the GGCTAA motif, arranged as double and inverted sites (separated by 10–12 bp) but also with a single GGC(A/T)_3_ motif [108, 109]. Interestingly, this last binding motif is present as single site in upstream region in all the XYR1-regulated genes [109]. While XYR1 appears to dimerize before binding to the DNA, dimerization is not essential for function, and it appears that posttranslational modification of XYR1 is the major mechanism governing (hemi)cellulase genes expression [106]. In addition to this, it has been shown that a strongly elevated basal transcription level of *xyr1* [69] and/or mutation in its regulatory domain [23] are responsible for the phenotype of *T. reesei* hyper-producer strains. In *T. reesei*, the shift between cytoplasm and nucleus is the key mechanism to switch the expression of target genes on/off. Under induction conditions, de novo biosynthesis and rapid nuclear import of XYR1 occur, while the termination of induction results in its rapid nuclear degradation [110]. This is similar to *A. niger*, where XlnR is inactive in the cytoplasm and nuclear import occurs under d-xylose induction [104].

Although XlnR orthologs are present in almost all filamentous Ascomycota, the set of genes controlled by this regulator is highly species specific [8] and appears to be linked to the lifestyle of the species. During evolution, it appears that this regulator had gradually lost functions, which led to adaptation to a more specialized biotype, as was observed in the pathogenic fungi (*Fusarium* spp. and *M. oryzae*) with the loss of cellulolytic function or in *Trichoderma* spp. [87] with the loss of mycoparasitism function from the “ancient” specie *T. atroviride* and the “most recent” *T. reesei* [87, 111]. Considering the composition of plant biomass (cellulose and hemicellulose are often present together) we can hypnotized that pathogenic fungi split the cellulolytic and xylanolytic function, most likely, to better control the host infection. In that sense, the saprobic *Talaromyces cellulolyticus* is a surprising exception, without an apparent biological explanation that deserves deeper investigation. Despite their taxonomic distance and different lifestyles, the functions of XlnR in *Fusarium* genus and *T. cellulolyticus* appear similar in that it regulates xylanase but not cellulase production [93].

#### l-Arabinose-responsive regulators (AraR and ARA1)

In *Aspergillus* spp., the arabinanolytic system is under control of AraR (homolog of XlnR), present only in the family Trichocomaceae. It appears to have originated from a gene duplication event from XlnR after this family split from the other filamentous Ascomycetes [112].

It has been reported that XlnR and AraR control distinct sets of genes in response to the presence of d-xylose and l-arabinose, respectively, but also interact with each other [112, 113]. Both regulators are involved in the regulation of genes encoding (hemi)-cellulases, as well as enzymes from the pentose catabolic pathway (PCP). and pentose phosphate pathway (PPP) [55]. In the absence of one regulator, the other can partially compensate for this loss [112]. The regulation of the PCP by AraR has been shown to differ inside the *Aspergillus* genus, both in expression and growth profiles, suggesting evolutionary changes in Aspergilli regarding pentose utilization [56]. Recently a new l-arabinose-responsive regulator, ARA1, has been described in *M. oryzae*. Despite having no significant sequence similarity to AraR, ARA1 appears to be the functional analog of AraR, controlling the arabinanolytic system as well as l-arabinose catabolism. A preliminary phylogenetic analysis shows that it is present only in the Sordariomycetes and Leotiomycetes (Additional file 2) and confirms that there is no significant sequence similarity to AraR [102]. This suggests that AraR and ARA1 are an example of parallel evolution in these fungal clades, which appears to be a relatively uncommon phenomenon for regulators in Ascomycetes.

#### *Neurospora crassa* cellulose regulators CLR-1 and CLR-2 and their homologs

Two transcription factors, CLR-1 and CLR-2, have been identified in *N. crassa* that are essential regulators of cellulolytic, but not hemicellulolytic gene expression [98]. Deletion of *clr*-*1* or *clr*-*2* is essential for growth and the complete loss of cellulase activity on cellulose, but not on xylan [98].

Homologs of these regulators are present in almost all genomes of filamentous Ascomycetes (Additional file 1). Despite differences in function have been reported, this prevalence suggests partially conserved regulatory mechanisms involved in cellulose utilization [98, 114]. These regulators have been fully characterized in *N. crassa* (CLR-1/2) and partially in *A. nidulans* (ClrA/B) [98, 115], *A. niger* [114], and *A. oryzae* (ManR) [116, 117], but only poorly in *Talaromyces cellulolyticus* (tCLB2) [118] and *Penicillium oxalicum* (CLR2) [25], showing both similarities and differences.

ClrA/1 appears to have a conserved role in cellulose-sensing, at least in *N. crassa* and *A. nidulans* [115]. The presence of cellulose or its products (i.e., cellobiose), results in the activation of ClrA/1, which induces expression of genes necessary for efficient import and utilization of cellobiose, including cellodextrin transporters and β-glucosidases, as well as *clrB/2*. Then ClrB/2 directly induces expression of cellulases in both fungi, while only a few selected hemicellulases are induced in *N. crassa* [98, 119].

The roles of these two TFs in other fungi seem to differ from what has been shown in *N. crassa*. Particularly in *Aspergillus* spp. ClrB (ManR) appears to have different functions compared to *N. crassa* CLR-2, and ClrA is not required for the induction of cellulases or other GH-encoding genes [114]. *T. reesei* lacks a clear homolog of CLR-1, indicating a different strategy for cellulase regulation/sensing in these species [120].

In *A. oryzae*, the CLR-2 ortholog was characterized as a regulator of mannan-degrading enzymes, and consequently named ManR [116]. Only later on, it has been shown to also control the cellulolytic system in this fungus [117]. Surprisingly, this protein is no longer present in the latest version of the *A. oryzae* genome and therefore not included in Additional files 1 and 2 in this study.

CLR-2/B is absolutely necessary for cellulolytic activity in *N. crassa, A. nidulans* [115], *A. niger* [114], *A. oryzae* [117], and *P. oxalicum* [25], but not in *T. reesei* [121] and *T. cellulolyticus* [118]. In fact in *T. cellulolyticus*, cellulase and xylanase production appears to be under control of the Zn_2_Cys_6_ TF TctA (*Fusarium ctf1B* homolog, involved in cutinase induction) [118], confirming a different regulation of (hemi)-cellulase in this species. Interestingly, in *N. crassa* CLR-1/2 regulates the expression of other main TF-encoding genes, necessary for the lignocellulose utilization, such as *xlr*-*1*, *vib*-*1*, *col*-*26* (*bglR* homolog; see “Amylolytic regulation: AmyR and MalR”), *sah*-*2* and *hac*-*1* involved in (hemi)cellulase production and *cpc*-*1* and the homolog of *tamA* involved in amino acid/nitrogen metabolism [119]. The opposite is the case in the Aspergilli, where ClrA and ClrB appear to be regulated by the main (hemi)-cellulolytic regulator XlnR [114].

The binding sites of CLR-1 and CLR-2 (Table 2) have been recently identified in *N. crassa*, as well as their mechanism. They act as a homocomplex and not as a heterocomplex as previously supposed [119]. Transcriptional analysis of *clr*-*2/B* mutants showed that only a relatively small group of cellulolytic genes have a strictly conserved dependence on CLR-2/B. This conservation between *Aspergillus* and *Neurospora*, which are only distantly related, suggests that this cellulase set is of major importance for cellulose utilization strategies in ascomycete filamentous fungi, but with a different regulatory mechanism [98, 115].

Recently Coradetti et al. [115] showed that it is possible to produce and secrete cellulases under noninducing conditions by misexpression of CLR-2 in *N. crassa,* but not of ClrB in *A. nidulans*. This could be explained by the fact that *N. crassa* CLR-2 is fully competent to drive transcription of its targets under noninducing conditions, while *Aspergillus* ClrB requires other factors, such as XlnR [51, 122], ClbR [17], or McmA [123, 124]. It suggests diversity in the signaling pathways that activate cellulases through XlnR and ClrB in filamentous fungi [98].

#### Specific activators of cellulase gene expression in *T. reesei*: ACE2 and ACE3

In *T. reesei*, the cellulase machinery is tightly regulated, involving other TFs in addition to XYR1: the repressor ACE1 [125] (see “Activator of cellulase expression 1 in *T. reesei* and hemicellulase regulator 1 in *N. crassa*”) and the two activators ACE2 [126] and ACE3 [121]. The latter two are classical Zn_2_Cys_6_ transcription factors, while ACE1 is a Cys_2_His_2_-type regulator (see “Activator of cellulase expression 1 in *T. reesei* and hemicellulase regulator 1 in *N. crassa*”).

ACE2 is acting as activator of cellulases and hemicellulases (mainly xylanases) in *T. reesei.* According to literature this regulator is considered as unique of the *Trichoderma* genus, but our phylogenetic analysis (Additional file 1) shows the presence of homologs also in some other Sordariomycetes. This suggests that *Trichoderma* spp., as well as few other Sordariomycetes, evolved a different regulatory system for (hemi)-cellulase production, compared to other species [3, 62].

Interestingly, ACE2 binds to the same promoter motif GGC(T/A)_4_ as XYR1 [109], but a second binding site GGGTAAATTGG was found in the xylanase-activating element sequence (XAE) in the *xyn2* promoter. It has been proposed that phosphorylation and dimerization are prerequisites for the binding of ACE2 to its target promoters [3].

When cellulose is the only carbon source, deletion of *ace2* in *T. reesei* resulted in the reduction of expression of all main cellulase-encoding genes (*cbh1*, *cbh2*, *egl1*, and *egl2*) and the xylanase-encoding gene *xyn2*, but not *xyn1*. This indicates that ACE2 acts as an activator of these genes, even though other factors also play a role in their induction. Sophorose induction is not affected by deletion of *ace2*, which suggests that the sophorose and cellulose inductions are at least partially mediated by different mechanisms [126]. Würleitner et al. [127] showed that in addition to ACE2, the HAP complex (HAP2/3/5) regulates *xyn2* expression in xylobiose-induced cultures, acting through the XAE sequence in the gene’s promoter.

Expression of *ace2* appears to be necessary for the formation of high levels of cellulase. *ace2* is induced by lactose and appears to not be affected by CCR, but similarly to XYR1, requires CRE1 for full induction during growth on lactose [69].

Recently, a novel regulator of lignocellulose degradation has been described in *T. reesei*: the activator of cellulase expression 3 (ACE3) [121]. Like ACE2, it is a typical Zn_2_Cys_6_ transcription factor and acts as positive regulator of cellulases and (partially) xylanases, both in direct and indirect manners, by regulating *xyr1* transcription. This suggests that *ace3* overexpression can both directly and indirectly through *xyr1* improve cellulolytic and xylanolytic gene expressions. For these reasons, it is considered a master regulator of cellulolytic and a modulator of xylanolytic genes [121].

Interestingly, our phylogenetic analysis (Additional file 2) shows that ACE3 is spread among the whole fungal kingdom (including Basidiomycota). Unfortunately it has not been characterized yet in other species to demonstrate its role in fungal plant biomass utilization.

#### Amylolytic regulation: AmyR and MalR

Two positive transcription factors have been reported to be involved in starch and maltose utilization in Aspergilli, AmyR and MalR, regulating amylase genes and maltose-utilizing (MAL) cluster genes, respectively [128]. AmyR is one of the first TF identified to be involved in plant biomass degradation in 1999 in *A. oryzae* [129], while MalR has been characterized more recently [128]. *AmyR* is well characterized in several *Aspergillus* species, such as *A. nidulans*, *A. oryzae* and *A. niger* [129–131]. Its orthologs were found in several Ascomycetes (Additional file 1), but its function has not been analyzed in these fungi.

Deletion of this regulator results in reduced or impaired growth on starch and/or maltose, due to insufficient production of the enzymes to convert these saccharides to d-glucose. *A. nidulans* AmyR shows 72 and 75% structural homology with its orthologs from *A. oryzae* and *A. niger*, respectively [132], and their DNA-binding domains are 100% identical to each other, which suggest that they recognize the identical DNA sequence CGGN_8_(C/A)GG with the A or C depending on the species. Most likely AmyR binds as a dimer with strong affinity to a motif with the two CGG triplets [133]. In addition, it binds also the CGGAAATTTAA sequence in amylase promoters in *A. oryzae* [134]. Two AmyR molecules are necessary to bind this sequence by recognizing the CGG triplet at the 5′-end and the AGG triplet just downstream of the sequence [134]. AmyR requires translocation to the nucleus to be activ). and the MH4 domain in the C-terminal region is essential to its cytoplasmic localization [135]. In fact, its deletion resulted in a constitutive nuclear localization of AmyR (and consequently it is constitutive active) in *A. nidulans* and *A. oryzae* [128]. However,, there are differences in the amylolytic regulation in these species. *A. niger* AmyR appears to be involved in the utilization of a broader range of oligo- and polysaccharides compared to other Aspergilli [131]. Deletion of a*myR* in *A. nidulans* results in no growth on maltose or starch, while in *A. oryzae* it only reduced growth on starch. The genes under control of AmyR appear to differ in these two strains [86, 131, 136], maybe due to the presence of the additional MAL cluster in *A. oryzae* [137]. This consists of a second maltose-responsive regulator (*malR*) [128, 137], a maltose permease (*malP*), and a maltase (*malT*) [128, 137]. This MAL cluster has been found in other, but not all Aspergilli, such as *A. fumigatus, A. flavus, A. clavatus*, and *A. fischeri* [138].

Deletion of *malR* in *A. oryzae* resulted not only in a growth defect on maltose and reduced *malP* and *malT* expression [137], but also in poor growth on starch and a reduced α-amylase activity on maltose but not on isomaltose [128]. Expression of *amyR* is induced by starch, maltos). and isomaltose (strongest), while *malR* in induced only by maltose and earlier in time than AmyR.

In contrast to AmyR, MalR is constitutively localized in the nucleus, probably due to the absence of the MH4 domain in its sequence [128]. All of this suggests that MalR is essential for the utilization of maltose and subsequent production of isomaltose as an inducer for AmyR activation in this fungus [128]. However, the mechanism of MalR activation is fully unknown and needs to be investigated deeper.

#### Glucose-sensing regulators: BglR and COL-26

BglR was initially identified in *T. reese*i as a regulator only of β-glucosidases genes with the exception of *bgl1*, which is seemingly under control of *xyr1* [139]. It is phylogenetically related to AmyR (Additional file 1).

Deletion of *bglR* increased cellulase production during cellobiose growth, possibly due the inability to produce a glucose signal for CCR. In *N. crassa*, the homolog of BglR, COL-26, regulates glucose sensing and metabolism, separately from CRE1-mediated CCR [99]. BglR is well conserved among filamentous Ascomycota, mainly in plant pathogen species which require cellulase production for a successful host infection, such as *Fusarium* spp., *Magnaporthe* spp., *Verticillium* spp., *Botrytis* spp., *Alternaria* spp., *Septoria* spp., etc. This suggests that BglR could be a main TF for those fungi which require a functional and accurate cellulose/glucose sensing for survival and/or virulence [139].

The *bglR* homolog in *T. cellulolyticus* (*tbgA*) does not control the cellulolytic system, but only affects part of the xylanolytic system. However, this needs to be confirmed by further investigation [118].

Much remains to be learned about BglR/COL-26 function, but it is likely to play a key role in cellulase overproduction in cellobiose cultures [139], mainly due to the involvement in glucose sensing [99].

#### ClbR: cellobiose response regulator

Recently, the TF ClbR has been described in *A. aculeatus*, which is involved in the early phase of cellobiose and cellulose induction through both XlnR-independent and XlnR-dependent signaling pathways [140]. It regulates *cbhI*, *cmc2*, *xynIa* (XlnR independent), *cmc1*, and *xynIb* (XlnR-dependent). However, all these genes are still induced in a Δ*clbR* strain, suggesting that others regulators, such as ClrA/B, are also involved [140] (see “*Neurospora crassa* cellulose regulators CLR-1 and CLR-2 and their homologs”). In fact, cellobiose-induction and XlnR-independent expression are under the control of both ManR (Clr2/B homolog) and ClbR [120].

Overexpression of ClbR in *A. aculeatus* resulted in an increase of a subset of xylanolytic and cellulolytic activities. In contrast, the cellobiohydrolase Cel7b was decreased, showing that the effects of ClbR overexpression are strictly depending on the type of enzyme. This suggests that ClbR is involved in diverse signaling pathways to regulate the expression of cellulose-degrading enzymes in *A. aculeatus* [17].

ClbR orthologs are only present in the Eurotiales, including *Aspergillus*, *Penicillium*, and *Talaromyces* species, while ClbR2 (ClbR paralog, 42% amino acid identity) orthologs are also found in other fungal orders and classes, including Sordariomycetes. Preliminary results in *A. aculeatus*, showed that ClbR2 regulates only *cbhI*, *cmc2*, and *manR*, and appears to compete with ClbR for the same DNA-binding region in the promoter of its target genes. ClbR2 is currently under investigation, in order to clarify its function, particularly in relation to ClbR [141].

Interestingly, some Eurotiales and Hypocreales (particularly *Trichoderma* spp.) genomes contain a third paralog with unknown function, named ClbR3 (Additional file 2; Fig. 2). The prevalence of these three TFs suggests that ClbR2 is the common ancestral TF from which ClbR and ClbR3 have originated, through (probably) duplication events after the divergence of those orders.

#### *Pectinolytic regulation: RhaR, GaaR, AraR*, and *GaaX*

The heterogeneity and complexity of the pectin structure suggests that regulation of pectinolytic gene systems could be much more complex than of the other polysaccharides, which is confirmed by the complex of regulators identified so far.

Wubben et al. [142] postulated that *Botrytis cinerea* endopolygalacturonases genes are regulated by four systems: basal expression, induction by pectin monomers, glucose repression and modulation by pH.

A similar model has been proposed in Aspergilli, where several TFs are involved and the expression profile of pectinolytic genes changes over time [70, 71, 143].

In *Aspergillus* spp., the system is controlled at least by AraR, responding to l-arabinose (see “The (hemi-)cellulolytic regulator XlnR/XYR1/XLR1”), RhaR, responding to l-rhamnose [37, 42, 144], and GalR/X, responding to d-galactose [113] (section “Inulinolytic regulation: InuR”) and the unknown ferulic acid regulator [143], while the d-galacturonic acid (GA) utilization is controlled by the novel activator GaaR [79] and the repressor GaaX [145].

*Aspergillus niger* RhaR controls the expression of the genes involved in l-rhamnose catabolism and only few pectinolytic genes, mainly related to the degradation of rhamnogalacturonan I [86]. The *RhaR* regulator is well distributed among Ascomycetes (phylogenetic see Additional file 1) [6], but it is not characterized yet outsides the *Aspergillus* genus. A novel d-galacturonic acid regulator has been recently characterized in *B. cinerea* (BcGaaR) [146] and *A. niger* (GaaR) [79]. It is essential for growth on d-galacturonic acid (GA), polygalacturonic acid (PGA). and (partially) pectins, regulating most of the pectinolytic genes, GA-transporters, as well as d-galacturonic acid catabolism. The residual growth reported in *A. niger* Δ*gaaR* during growth on pectin, can be easily explained by the presence of alternative regulation mechanisms independent of GaaR and/or by growth on other sugars from pectin, such as l-arabinose, d-galactose, d-xylose or l-rhamnose, which are regulate by different TFs (AraR, GalR/X, XlnR, RhaR etc.) [79]. This TF binds to the GARE motif (TCCNCCAAT) of the target genes in both species, and it is imported into the nucleus during induction, similarly to XlnR/XYR1 in *A. niger* and *T. reesei* (see “The (hemi-)cellulolytic regulator XlnR/XYR1/XLR1”). In addition to this, in *A. niger* the repressor GaaX appears to inactivate GaaR in the absence or at low levels of intracellular GA, ensuring a rapid response to the presence of GA as it does not require de novo synthesis of GaaR [145].

Homologs of GaaR are present in most filamentous Ascomycetes, including industrially relevant genera, such as *Aspergillus* spp. and *Trichoderma* spp. (Additional file 2). *GaaX* is located next to *gaaR*, and this organization appears to be conserved in most of Ascomycota species analyzed [145], suggesting that also the regulation of GA utilization is conserved across filamentous Ascomycota.

#### Inulinolytic regulation: InuR

InuR [147] appears to be a homolog of AmyR, and they likely have originated from a common ancestor [37, 43]. Additional support for this is that the InuR putative DNA-binding site (CGGN_8_CGG) seems to be identical to the DNA-binding site of AmyR [43]. Only when both *inuR* and *amyR* are deleted growth is abolished on inulin in *A. niger*, suggesting coregulation by these regulators of inulin conversion [147]. InuR regulates inulinolytic genes (*inuA* and *inuE*) and sucrose metabolism (*sucA* and *sucB*), as well as the (putative) transporters related to these sugars. Our phylogenetic analysis (Additional file 1) shows that it is mainly present in Aspergilli and related species. *F. oxysporum* appears to have four homologs of InuR, consisting of two pairs of identical TFs, suggesting different functions in this fungus.

#### d-Galactose-responsive regulators: GalR and GalX

So far, d-galactose-responsive regulators have been described only in aspergilli (GalX and GalR) and yeasts, e.g., *Saccharomyces cerevisiae* (Gal4, Rtg1 and Rtg3) [9]. Those TFs do not appear to have a common ancestor (Additional file 1) as they share low sequence homology [148]. GalX and GalR share only 12% amino acid identity, while GAL4 shares 11% aa identity with GalR and 20% with GalX. Moreover, high variation within their DNA binding domains has been reported [148]. In agreement with this, differences in d-galactose utilization have been observed among the Aspergilli and even more between aspergilli and *S. cerevisiae* [148–150].

GalX is present in the whole genus, regulating the oxido-reductive d-galactose catabolic pathway [151], while GalR is only present in section *Nidulantes* (e.g., *A. nidulans, A. versicolor*, *A. sydowii* [113], controlling the Leloir pathway. So far no regulators have been reported to control this pathway in other Aspergilli. Interestingly, GalR appears to be under control of GalX in *A. nidulans*, and it has been shown that in this fungus d-galactose catabolism is not only regulated by this two TFs, but also includes involvement of XlnR and AraR [113]. Considering that d-galactose, l-arabinose and d-xylose are often present together in nature, this coregulation is not surprising.

Although both GalX and GalR have so far been mainly shown to be involved in d-galactose catabolism, there are some indications for an involvement in d-galactose release from polysaccharides [113]. Particularly GalR appears to trigger the expression of one α-galactosidase (AGL). However, for full control of this gene, cooperation with AraR is required [151].

Phylogenetic analysis (Additional file 1) showed that GalX and GalR cluster separately, disclaiming the possibility of gene duplication and no orthologs are present outside this genus. In other species, the presence of specific d-galactose-responsive regulator(s) is not clear yet and currently under investigation, particularly in *T. reesei* [61, 152]. In this fungus, a lactose/d-galactose response has been reported that is mediated by XYR1 and other(s) unidentified TF(s), possibly including CLR2 [67, 68]. This suggests that most likely a network of TFs is involved instead a single specific TF in this fungus [68].

Differences in growth on d-galactose and its regulation have been reported among Aspergilli, suggesting a different evolution related to d-galactose utilization in this genus. For example, *A. niger* cannot grow on d-galactose from spores, due to the absence of d-galactose uptake during germination [153], while *A. nidulans*, *A. sydowii* and *A. versicolor* can use this sugar as a sole carbon source (http://www.fung-growth.org). This suggests that GalR (present in these species, but not in *A. niger*), may be required for expression of genes encoding d-galactose transporters during germination [113].

### Cys_2_His_2_ transcription factors

#### Carbon catabolite repression: CreA/1

Carbon catabolite repression (CCR) is a universal regulatory system which prevents wasting energy on the production of extracellular enzymes, as well as metabolic routes that are not needed. In relation to plant biomass conversion, this means that when there is sufficient monosaccharide already present, there is no need to produce enzymes to release more monomers from the polysaccharides, and therefore CreA/CRE1 represses the expression of the genes encoding these enzymes [62, 154–156]. Sugar sensing (mainly coordinated via cAMP-dependent PKA pathway and phosphorylation of glucose) induces CCR which repress the utilization of alternative carbon sources [157]. In fungi, CCR occurs mainly through the well conserved Mig1/CreA/CRE1/Cre1 Cys_2_–His_2_ double zinc finger TF. So far this TF is the only one which is conserved throughout the fungal kingdom (Additional file 3), suggesting a conserved mechanism for CCR in fungi. It is well studied in *S. cerevisiae* and filamentous Ascomycota, particularly in *Aspergillus* spp., *T. reesei* and *N. crassa,* but not yet in Basidiomycota.

So far all binding sites of CreA/1 described are two closely spaced SYGGRG, and repression occurs only through this double-binding sites. However, data from in vitro studies suggested that only one site is required, as has been shown in *cbh2* promoter in *T. reesei* [158].

In general, deletion of *creA/1* leads to derepression of transcription of (hemi)-cellulolytic genes and other genes involved in polymeric carbon sources, under both repressing and inducing conditions [69, 155, 159, 160].

In aspergilli and *N. crassa*, CreA/1 directly represses mainly expression of the genes encoding enzymes involved in plant cell wall degradation, such as (hemi)-cellulases [159, 161] or the TF(s) involved in this process [160, 162], while this has not been observed in *T. reesei*. In this fungus, CRE1 appears to mainly “switch on/off” the transport of inducers/repressor depending on the growth conditions [45, 163, 164] and consequently the TFs which should trigger the expression of the (hemi)-cellulolytic systems.

Although all main TFs, including *xlnR/xyr1/xlr1*, appear to be under control of CCR and have CreA/CRE1 and/or ACEI (see “Activator of cellulase expression 1 in *T. reesei* and hemicellulase regulator 1 in *N. crassa*”) binding sites in their promoter region, evidence of direct regulation only exists for a few cases. A direct effect has been reported for *xlnR/xyr1* in *A. nidulans* [162], *A. niger* [59], *T. reesei* [106, 155, 163] and *T. koningii* (208), with *ace2* and *ace1* in *T. reesei* [69], and with *amyR* in *A. nidulans* [136, 165] and *N. crassa* [159].

However, during repressing condition in *T. reesei* [106] and *T. koningii* [166], the main target of CRE1 is XYR1, while in *N. crassa* XLR-1 appears to be regulated mainly by other CCR mechanisms [88]. Moreover, in *T. reesei* CRE1 acts together with other regulatory proteins in a coordinated manner to promote CCR and optimize C-source utilization. This ensures a good adaptation to different growth condition [163].

*Trichoderma reesei* appears to be the only fungus in which full induction of *xyr1* and *ace2* requires the positive action of CRE1, at least during growth on lactose [69]. In *T. reesei* RUT-C30, partial truncation of CRE1 (CRE1-96, which lacks one zinc finger) leads to an increase of cellulase production, while a full deletion has a less pronounced effect [167]. CRE1-96 controls a more open chromatin at three levels: direct action on the promoters of its target genes, and in an indirect way by increasing the transcription of a chromatin-remodeling protein HTF1. The third level is the loss of the autoregulatory function of CRE1, leading to high transcript levels of *cre1*-*96* [167]. RUT-C30 is an industrial cellulase hyper-producer strains obtain by several rounds of random mutagenesis [21], and is still under investigation to clarify what exactly enhances cellulase production.

Beside transcriptional regulation, CCR appears to act also through chromatin remodeling. In *A. nidulans* and *T. reesei* CreA/1 directly affects chromatin structure (packaging, nucleosome position, acetylation etc.) during repression condition. Particularly, *T. reesei* CRE1 is involved in organizing the local chromatin structure (packaging) or nucleosome positioning in *xyr1* promoter and cellulases *cbh1* and *cbh2* during repressing conditions, and its loss leads to a less dense chromatin structure during CCR [168–170]. Similarly to this, in *A. nidulans* CreA is involved in chromatin remodeling, through histone deacetylation [171].

A CreA/1 deletion also results in impaired colony morphology in almost all analyzed fungi [159, 172–174], such as smaller and more compressed colonies, with fewer aerial hyphae and spores. In addition, no deletion strains have been obtained in *F. oxysporum*, *P. chrysogenum* and *M. oryzae*, suggesting that this mutation is lethal in these species [175]. The only exceptions to this impaired morphology in deletion strains are *Alternaria citri* and *Alternaria brassicola*, suggesting that this genus regulates expression of cell wall-degrading enzymes in a novel manner [176, 177].

CreA/1 is also involved in many other processes, such as penicillin production in *P. chrysogenum* [178], mycoparasitism in *T. harzianum* [179], nitrogen and amino acid transport/metabolism in *A. nidulans* and *T. reesei* [160], and environmental pH control in pathogenic fungi [175]. All these suggest that CreA/1 have broader function then initially known.

The mechanism of CCR is more complex in filamentous fungi compared with *S. cerevisiae*, reflecting the differences in lifestyle and the ability of filamentous fungi to utilize a broad range of C-sources, such as pentoses.

In contrast to *S. cerevisiae*, CCR in filamentous fungi is not only regulated by glucose, but also by high concentrations of other monosaccharides and by nuclear localization [156]. In *Sclerotinia sclerotiorum* [180] and *T. reesei* [110], nuclear localization of Cre1/A is glucose dependent, while in *A. nidulans,* this is not the case [181]. Also the transcription level of *cre1/A* differs between species. In *T. reesei* and *A. nidulans, cre1/A* are negatively autoregulated and transcribed at lower level in repressing conditions [110, 182, 183], while in *Acremonium chrysogenum, cre1* transcription is dependent on glucose concentration [184]. In *B. cinerea* and *Gibberella fujikuroi, creA* expression is C-source independent [185].

Moreover, in filamentous fungi, carbon catabolite derepression occurs only in the presence of inducers and during carbon limitation or metabolic stress [156], while it is not so in the case in *S. cerevisiae*. In addition to d-glucose, high concentrations of other simple C-sources trigger CCR through nuclear localization of CreA/1 in filamentous fungi, such as cellobiose and xylose in *A. nidulans,* or ethanol in *F. oxysporum* [156].

In both *S. cerevisiae* and filamentous fungi, phosphorylation of CreA/1, mediated by SnfA/1, is a key for its re-localization during derepression conditions. In *A. nidulans* SnfA is essential for removal of CreA from the nucleus [186], but not in *S. sclerotiorum*, although it is still necessary for Cre1 derepression [187]. In contrast to other lignocellulolytic fungi, in *T. reesei*, CRE1 is positively regulated by phosphorylation under repressing conditions [188]. Moreover, SNF1 (SnfA orthologs) is not able to phosphorylate CRE1 in vivo in *T. reesei*, while it is able to phosphorylate Mig1 when heterologously expressed in yeast. These differences, suggests that CCR has evolved differently in *T. reesei* compared to other fungal species [156].

In *A. nidulans, A. oryzae*, and *T. reesei*, CreA/1 forms an essential complex for CCR with the deubiquitinating enzyme CreB/2 [189–191], the WD40 motif-containing protein CreC [192] and HECT-type ubiquitin ligase-interacting protein CreD [181, 193]. These additional proteins influence CreA/1 stability and proteosomal degradation, as well providing a link between ubiquitination and phosphorylation in protein regulation and stability. In *A. nidulans* [194] and *F. oxysporum* [195], F-box proteins (particularly the unique fungal protein FbxA) also are involved in hemi-cellulase production during CCR. In conclusion, carbon catabolite repression in fungi is not only regulated by CreA/1, but F-box, and additional-Cre proteins introduce a new level of complexity to this system [156].

#### Activator of cellulase expression 1 in *T. reesei* and hemicellulase regulator 1 in *N. crassa*

In contrast to its initially assigned name, in *T. reesei* and *T. koningii*, ACE1 acts as a repressor for both cellulase and xylanase production [106, 125, 196, 197]. In contrast, in *Talaromyces cellulolyticus*, its homologous gene *tacA* appears to act as an inducer of cellulases and xylanases, as well as cutinases. However, the TacA protein shows a low similarity to ACE1 except for the zinc finger domain (this is confirmed also from BlastP analysis, data not shown), suggesting that it is a novel transcriptional regulator protein [118]. Homologs of ACE1 have been found in almost all filamentous Ascomycota, but very few have been characterized.

The ACE1 protein contains three Cys_2_His_2_-type zinc fingers and was shown to bind in vitro to eight sites in the *cbh1* promoter, all of which contain the core AGGCA sequence [197]. This core sequence is found in nearly all cellulase promoters in *Trichoderma* spp., but their functions are not completely clear. In the *xyn1* promoter, ACE1 binds to two GGCTAA motifs, competing with the positive regulator XYR1 [108].

In sophorose- and cellulose-induced cultures of *T. reesei*, the *ace1* deletion resulted in an increase in the expression of genes encoding all the main cellulases (such as *cbh1*, *cbh2*, *egl1*, and *egl2*) and hemicellulases (such as *xyn1* and *xyn2*) [197]. Despite its repressor role, *ace1* transcription is induced by lactose, and repressed by CRE1-mediated CCR [69].

All *ace1* deletion strains show strong impaired growth on d-sorbitol, suggesting additional targets and a more general regulatory role than expression of cellulase- and hemicellulose-encoding genes [62, 197]. In *A. nidulans*, a gene (*stzA*) encoding a highly similar protein to ACE1 is involved in abiotic stress response, such as sensitivity to salt and DNA damaging agents [198]. According to our phylogeny, this appears to be a clear example or divergent evolution of a TF (Additional file 3).

Recently in *N. crassa* a novel Cys_2_His_2_-type zinc fingers TF involved in hemicellulase regulation, HCR-1, has been characterized [199]. This regulator acts as a hemicellulase repressor (affecting mainly xylanases) during growth on l-arabinose or xylan. It appears to be conserved across several cellulolytic fungi but not characterized yet in other species, suggesting a conserved role in lignocellulose degradation. Unfortunately, very little is currently known about its function(s), and deeper investigations are required, particularly regarding its mechanism and role in the regulatory network [199].

### Transcription factors without zinc finger: MADS-box

#### McmA

MADS-box proteins are a well conserved family of transcription factors in eukaryotic organisms, controlling a broad range a cellular function through interaction with their cofactors, such as primary metabolism, cell cycle, and cell identity. Based on the amino acid sequence of the conserved MADS-box domain, these protein are classified into two types: SRF-like (Serum Response Factor) (type I) and MEF2 (Myocyte Enhancer Factor2) (type II). Only few MADS-box proteins have been reported in fungi, mainly in the SRF-like subfamily [200].

The MADS-box motif, of SRF proteins, generally binds to the consensus sequence CC(A/T)_6_GG (the CArG box) on the target genes [201, 202]. The MAD-domain is also responsible for the nuclear localization, DNA-binding specificity, accessory factor binding and dimerization of the protein [200, 203].

In *A. nidulans*, the MADS-box protein McmA (similar to *S. cerevisiae* Mcm1) has been shown to positively control cellulase expression, probably through interaction with ClrB/ManR [120, 124]. McmA and ClrB both bind to the cellulose-responsive element (CeRE) of the endoglucanase *eglA* promoter in a cooperative manner [124], confirming the common claim [201, 204, 205] that MADS-box proteins have a strong ability to interact with others proteins, integrating different biological processes. In contrast, in *T. cellulolyticus,* the *mcmA* homologous *tmcA* appears to have only small effects on cellulase production [118].

## Other factors affecting plant biomass utilization

Additional factors affect plant biomass utilization by fungi, such as temperature, pH, light, nitrogen sources, and the access to heterochromatin. These phenomena are poorly studied in relation to plant biomass utilization, but are crucial to fully understand the regulation of this process. In addition, their signal pathways are not clarified yet, and most likely, they are involved in the transduction of many different processes. Here we present a short overview of these factors.

### Xylanase promoter-binding protein (XPP1)

Recently a novel transcription factor has been characterized in *T. reesei*, named xylanase promoter-binding protein (XPP1) [206]. It acts as a repressor for xylanase-encoding genes (*xyn1*, *xyn2*, and *bxl2*) during growth on d-glucose or high d-xylose concentration at later cultivation stages, while it has no effect on cellulase or d-xylose catabolism [206]. In addition, recently it has been shown that XPP1 is a switch between primary and secondary fungal metabolism (mainly repressing secondary metabolism), suggesting that its role in regulating xylanases may just be a secondary effect [207].

XPP1 is a basic helix-loop-helix TF (bHLH) with E-box domain, which typically would bind to a hexameric palindrome 5′-CANNTG-3′ [208]. Experimental observations suggest that most likely the actual binding is composed of the hexameric palindrome 5′-WCTAGW-3′ together with an inverted AGAA-repeat [206]. Phylogenetic analysis shows that this regulator is only present in Sordariomycetes (Additional file 5).

### Chromatin access

Access to heterochromatin is involved in the control of gene expression and therefore also in plant biomass utilization. It is mainly organized through the CCAAT box (Hap complex), methylation (mainly through LaeA/1 [209–212]). and acetylation levels. The CCAAT box complex is believed to be necessary for the generation of an open chromatin structure, which enables full transcriptional activation of certain promoters [158, 213].

CCAAT sequences are present in the 5′ regions of approximately 30% of all eukaryotic genes. The Hap complex is the first CCAAT-binding complex described in *Saccharomyces cerevisiae*, and it consists of the Hap2, Hap3, Hap4, and Hap5 proteins. Homologs have been identified in several organisms, such as HAP2-3-5 in *T. reesei*, Hap5 in *N. crassa* and AnCF from *A. nidulans* [214].

CCAAT sequences have been found not only in the promoters of respiratory genes [154], but also in promoters of many cellulase- and hemicellulase-encoding genes as well as in the promoters of the ligninolytic genes of several fungi. This sequence in promoter regions appears to be essential for gene expression, as was reported for *cbh2* in *T. reesei* [215]. This reduction in gene expression occurred either at the basal level or in response to specific induction signals, indicating that the CCAAT motif cooperates with other specific elements to affect transcription [216].

An opposite result was obtained when the CCAAT sequence in the XAE in the *T. reesei xyn2* promoter was mutated, resulting in a slight increase in *xyn2* transcription in glycerol and xylan grown cultures [127]. Moreover in *T. reesei* the HAP complex, CRE1 and an unknown GTAATA-binding protein affect nucleosome positioning, influencing the accessibility to the TATA box for transcription initiation of *cbh2* [158]. More recently and confirming this, Cre1/A has been shown to directly affect the chromatin structure (packaging, nucleosome position, acetylation etc.) during repression condition in *A. nidulans* and *T. reesei* (see “Carbon catabolite repression: CreA/1”) [168, 171].

Acetylation levels influence the access to chromatin and consequently the expression of genes. In *T. reesei* the histone acetyltransferase GCN5 has been deleted, which is involved in chromatin modification by catalyzing the acetylation of specific lysine residues within the N-terminal tails of the core histones. This deletion severely affects acetylation levels resulting in impaired growth, morphogenesis, and expression of cellulase-encoding genes [217].

### Light effect

Filamentous fungi can rapidly react to light [218–221], affecting morphology, development and primary and secondary metabolism [222–226], as well as sexual and asexual reproduction [227, 228].

Consequently light influences the lignocellulose degradation in many fungi, such as *T. reesei* [229–232], *T. atroviride* [233, 234], *N. crassa* [119, 235], and Aspergilli [236–238], regulating at the (post)-transcriptional level sugar uptake, carbon catabolism and production of hydrolytic enzymes [223, 239]. For example, in *T. atroviride*, it has been shown that light strongly influences growth on diverse C-sources [240].

Two light-responsive complexes have been described so far in fungi. The White collar complex (WCC) [239, 241] together with VIVID (VVD) [218] mainly responds to blue light/UV-A through photoreceptors WC1 and WC2 (*N. crassa*) (BLR1, BLR2 in *T. reesei*) [219, 235]. These photoreceptors regulate metabolic pathways in response to light [242] in *T. reesei* [219] and *N. crassa* [235]. In *N. crassa*, it has been shown that WWC controls several TF depending on circadian rhythms (Dawn- and dusk-phases), including the glucose-dependent repressor CSP1, important for (hemi)-cellulase production [243]. The other system is the heterotrimeric VELVET complex [211, 224], consisting of VeA—the velvet-like protein VelB—and LaeA, which controls secondary metabolism as well as sexual and asexual reproduction in several species [212, 224, 244–246]. Interestingly, *T. reesei* and *T. atroviride* LAE1 (LaeA ortholog) and VEL1 (VeA ortholog) are essential for the expression of (hemi)-cellulase-encoding genes [211, 233, 247], while this was not observed in other species, such as Aspergilli [248, 249].

These light-responsive systems are in general highly conserved in Ascomycetes, but some species do not have VVD orthologs, such as *Aspergillus* spp. [236], indicating that they are also differentially organized [219].

These data confirm the crosstalk between nutrition, circadian clock and light response in filamentous fungi [119, 220, 231, 240].

### TFs involved in nitrogen and pH regulation

Plant biomass utilization is also affected by nitrogen sources, environmental pH and Reactive Oxygen Species (ROS).

Similarly to CCR, Nitrogen Metabolite Repression (NMR) enables preferential utilization of easily assimilated N-sources (ammonium or glutamine) instead of energetically less-favored ones (i.e., nitrate), preventing wasting energy [250, 251]. This mechanism will also negatively affect hydrolytic enzyme production during nitrogen starvation, as well as carbon and secondary metabolism, in several fungi, such as *A. nidulans* [252, 253], *Trichoderma* spp. [234, 254], and *N. crassa* [50, 255–257] or *Fusarium* spp. [250]. This effect is well studied in *A. nidulans* and *N. crassa*, and occurs through 3 key TFs: the activator AreA/NIT-2 (*A. nidulans* and *N. crassa*, respectively) [253, 257], and two repressors AreB [258] and NmrA/1 [259, 260].

AreA belongs to GATA family with Cys_2_Cys_2_-binding-domain [261], and it is considered the main general nitrogen status-sensing regulator: under N-limiting condition or starvation activates a broad range of genes involved in the utilization of alternative N-sources, such as catabolic genes and permeases [262]. AreA activates directly the transcription of target genes, stimulates the pathway-specific TF(s) necessary to metabolize only the “secondary” N-source present (i.e., NirA [263] in the presence of nitrate) [264], and remodels the chromatin [265] and increases histone acetylation [252]. The activity of AreA is modulated at the post-transcriptional/translational level by its corepressor NmrA through direct binding during N-sufficient conditions [259, 266].

Another GATA repressor is AreB, coding for three distinct proteins [258]. It modulates AreA activity, repressing Area-dependent nitrogen catabolic genes under C-limiting conditions, probably through DNA-binding competition [267]. In addition, it acts also pleiotropically, regarding growth, conidial germination and asexual development [268].

Macios et al. [267] showed that these 3 main TFs negatively regulate arginine catabolism (used as N and C-source) in *A. nidulans*. In this system, NmrA appears to modulate AREA and AREB activities in response to the carbon status of the cell [267], confirming the crosstalk between Carbon and Nitrogen regulation proposed by Lockington et al. [269].

This crosstalk can explain the negative effects of *N*-starvation on hydrolytic enzyme (CAZy) production observed and described above. Those regulators mediate chromatin remodeling, in particular during nitrogen starvation, affecting the CAZyme production in *A. nidulans* [250, 252]. Particularly AreA appears to have a major role in this and cellulase-encoding genes in *A. nidulans* contain potential binding sites for the global carbon and nitrogen regulatory TFs, such as CreA, XlnR and AreA, in their promoter [269]. This supports the existence of a link between regulation of carbon and nitrogen metabolism in fungi [269].

Fungi also need to adapt to ambient pH changes [270], mediated through an elaborate signal transduction network to allow the proper physiological response, such as production of specific hydrolytic enzymes, growth, cellular transport, development, and pathogenicity/virulence [271–277].

The pH regulation has been well studied in *A. nidulans* [271, 278] and *Saccharomyces cerevisiae* and reviewed previously [279]. In summary, the fungal response to pH occurs through the Pal signal-sensing pathway and the key TF PacC (Cys_2_His_2_ type), both highly conserved in fungi [271]. The active form of PacC (PacC^27^) induces the transcription of alkaline-expressed genes (including *pacC* itself) and represses transcription of acid-expressed genes [280, 281], including lignocellulolytic enzymes. Recently, another factor involved in pH response has been described: the transcription factor PacX [282]. This is a repressor of PacC, but its exact mechanism and function are not clear yet. In addition, PacC not only regulates (hemi)-cellulase production directly by binding the promoter regions of the corresponding genes, but mainly indirectly via modulation of the activity of the transcriptional regulators involved in their production, such as XlnR [283] and ClrB [284] or/and uncharacterized TF(s) as shown in *T. reesei* [285]. Particularly in *T. reesei* PAC1 (homolog of PacC) increases cellulase production by induction of *xyr1* and *ace2* expression [276]. This indicates that the pH-regulation, besides its conservation across fungi, may differ depending on the species.

## Conclusion

Due to the complexity of plant biomass, fungi have evolved different strategies to utilize all or some of its components as carbon source. They need a broad set of degrading enzymes as well as diversified metabolic pathways, which are under control of several regulators. An additional level of complexity is the crosstalk between regulatory systems. Moreover, regulatory systems are poorly conserved in fungi. This is likely caused by the need of different fungal species to adapt to their specific biotope such as forest, crops, herbivore dung, and gut etc. On top of that, the lifestyle of every fungus (such as saprobe, pathogen, symbiont, etc.) adds a second level of adaptation, affecting gene regulation. Example of this complex adaptation is the transcriptional rewiring, where a single TF has different functions depending on the species.

With the availability of an ever-increasing number of fungal genomes, transcriptomes, and proteomes, the differences between regulatory systems in fungi related to in plant biomass utilization have become more evident. This study demonstrated both similarities and differences between fungal species, as well as the much higher level of complexity of and interaction between the regulatory systems than was previously assumed. With the addition of new regulators, our understanding of plant biomass strategies of fungi increases, enabling a more fine-tuned manipulation of enzyme production in industrial settings. This will not only benefit the development of better enzyme cocktails for the production of biofuel and biochemical, but also many other industrial sectors, such as pulp and paper, food and feed, and textiles.

## Additional files

**Additional file 1.** Prevalence of transcription factors in Fungi.

**Additional file 2.** Maximum likelihood tree of Zn_2_Cys_6_ transcription factors (TFs). In bold are the species for which the TF has been characterized, while in bold and larger font are the species in which the TF was first discovered.

**Additional file 3.** Maximum likelihood tree of Cys_2_Hys_2_ transcription factors (TFs). In bold are the species for which the TF has been characterized, while in bold and larger font are the species in which the TF was first discovered.

**Additional file 4.** Maximum likelihood tree of MADS BOX (McmA) transcription factors (TFs). In bold are the species for which the TF has been characterized, while in bold and larger font are the species in which the TF was first discovered.

**Additional file 5.** Maximum likelihood tree of bHLH (XPP1) transcription factors (TFs). In are the species for which the TF has been characterized, while in bold and larger font are the species in which the TF was first discovered.

## Abbreviations

TF: transcription factor
CAZy: Carbohydrate-Active enZymes database
HGA: homogalacturonan
XGA: xylogalacturonan
RG: rhamnogalacturonan
GH: glycoside hydrolases
GT: glycosyltransferases
PL: polysaccharide lyases
CE: carbohydrate esterases
AA: auxiliary activities
CBM: carbohydrate-binding modules
bHLH: basic helix-loop-helix
HECT: homologous to the E6-AP carboxyl terminus
WD40: beta-transducin repeat
CCR: carbon catabolite repression
XAE: xylanase-activating element
ROS: reactive oxygen species
SRF: serum response factor
MEF2: myocyte enhancer factor2
CeRe: cellulose-responsive element
WCC: white collar complex
VVD: VIVID complex
cAMP: cyclic AMP
PKA: protein kinase A
PCP: pentose catabolic pathway
PPP: pentose phosphate pathway
ABF: arabinofuranosidase
BXL: β-xylosidase
XYN: xylanase
CBH: cellobiohydrolase
EGL: endoglucanase
BGL: β-glucosidase
CMC: carboxymethyl cellulase
AGL: α-galactosidase
INU: inulase
