# Supplementary material for: Regulators of plant biomass degradation in ascomycetous fungi
Source: Biotechnol Biofuels. 2017 Jun 12;10:152. doi: 10.1186/s13068-017-0841-x (PMC5468973; doi:10.1186/s13068-017-0841-x)
Supplement: Supplementary file 2 — Additional file 2. Maximum likelihood tree of Zn2Cys6 transcription factors (TFs). In bold are the species for which the TF has been characterized, while in bold and larger font are the species in which the TF was first discovered. [file 13068_2017_841_MOESM2_ESM.pdf]

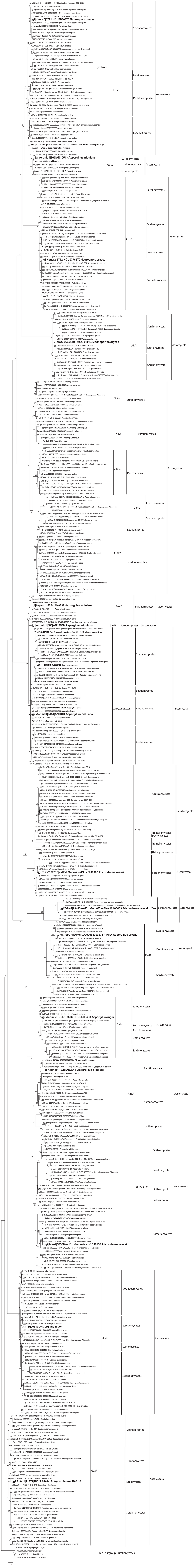

Additional File 2. Maximum likelihood tree of Zn_2Cys_6 Transcription Factors (TFs).
In bold are the species for which the TF has been characterized, while in bold and larger font are the species in which the TF was first discovered.
